# Impact of cultivar selection and process optimization on ethanol yield from different varieties of sugarcane

**DOI:** 10.1186/1754-6834-7-60

**Published:** 2014-04-12

**Authors:** Yuda Benjamin, Maria P García-Aparicio, Johann F Görgens

**Affiliations:** 1 Department of Process Engineering, Stellenbosch University, Private Bag X1, Matieland, Stellenbosch 7602, South Africa

## Abstract

**Background:**

The development of ‘energycane’ varieties of sugarcane is underway, targeting the use of both sugar juice and bagasse for ethanol production. The current study evaluated a selection of such ‘energycane’ cultivars for the combined ethanol yields from juice and bagasse, by optimization of dilute acid pretreatment optimization of bagasse for sugar yields.

**Method:**

A central composite design under response surface methodology was used to investigate the effects of dilute acid pretreatment parameters followed by enzymatic hydrolysis on the combined sugar yield of bagasse samples. The pressed slurry generated from optimum pretreatment conditions (maximum combined sugar yield) was used as the substrate during batch and fed-batch simultaneous saccharification and fermentation (SSF) processes at different solid loadings and enzyme dosages, aiming to reach an ethanol concentration of at least 40 g/L.

**Results:**

Significant variations were observed in sugar yields (xylose, glucose and combined sugar yield) from pretreatment-hydrolysis of bagasse from different cultivars of sugarcane. Up to 33% difference in combined sugar yield between best performing varieties and industrial bagasse was observed at optimal pretreatment-hydrolysis conditions. Significant improvement in overall ethanol yield after SSF of the pretreated bagasse was also observed from the best performing varieties (84.5 to 85.6%) compared to industrial bagasse (74.5%). The ethanol concentration showed inverse correlation with lignin content and the ratio of xylose to arabinose, but it showed positive correlation with glucose yield from pretreatment-hydrolysis. The overall assessment of the cultivars showed greater improvement in the final ethanol concentration (26.9 to 33.9%) and combined ethanol yields per hectare (83 to 94%) for the best performing varieties with respect to industrial sugarcane.

**Conclusions:**

These results suggest that the selection of sugarcane variety to optimize ethanol production from bagasse can be achieved without adversely affecting juice ethanol and cane yield, thus maintaining first generation ethanol production levels while maximizing second generation ethanol production.

## Background

Sugarcane represents a preferred crop for the production of bioethanol, which is the most widely used biofuel in the world today, due to high biomass yields and high fermentable sugar content [1,2]. Integration of first and second generation technologies for ethanol production from both sugarcane juice and the lignocellulosic residue (bagasse) could improve the sustainability and economics of the process, thereby increasing the ethanol yield per ton of harvested sugarcane [3]. However, the recalcitrance of the lignocellulose requires a more complex processing technology when compared to the juice, in order to obtain the fermentable sugars. The biochemical production of ethanol from lignocellulose involves the subsequent steps of pretreatment, enzymatic hydrolysis and fermentation. Although numerous advances have been made towards cellulosic ethanol in the last few decades, its production at large scale is still hampered by pretreatment and enzyme costs [4,5]. Reduction of production costs can be obtained through optimization of the different steps in an integrated manner, since each step has an impact on the next [3,6]. The direct use of pretreated material at high solids loading as substrate during simultaneous saccharification and fermentation (SSF) is considered a promising strategy to reach at least 4% volume by volume (v/v) ethanol in the fermentation broth [7]. Problems associated with inhibitors and mixing at high solids loading can be alleviated by using the pressed pretreated material and fed-batch feeding during the SSF [8].

One aspect that has received less attention is the impact of feedstock properties on the operational conditions and economics of the production process. It has been demonstrated that variations in feedstock lead to different process requirements, even for similar biomass or varieties of the same species [6]. Therefore, further reduction of global cost could be obtained through crop development and selection of varieties with advantageous traits including agronomic properties (high biomass, sugar and fiber production per hectare) and, in the case of lignocellulosic residues, being more amenable to conversion to monomeric sugars through pretreatment-hydrolysis, often related to high structural carbohydrates content, reduced lignin content and improved digestibility. These aspects are referred to as ‘feedstock quality’ in the present study.

Biomass yield and composition, and ultimately sugar and ethanol yields, vary depending on various factors such as variety (genotype), year, harvest period and location [9,10]. Several studies have proven the negative correlation between cellulose digestibility with lignin and ash contents, whereas it is improved by carbohydrate content [11-14]. A selection of varieties with fibers with a high ratio of carbohydrate: lignin and reduced ash content would be beneficial to maximize sugars and ethanol yield, provided that other agronomic traits are not compromised. Up to 26% difference in the sugar yields were observed from the straw of different cultivars of wheat when applying a standard hydrothermal pretreatment followed by enzymatic hydrolysis [14]. Similar differences have been found for feedstocks with reduced lignin content in ethanol yield during SSF of alkali-pretreated corn stover [15] and dilute acid pretreated sorghum bagasse [12]. A more recent work evaluated the impact of the genotype of maize on sugar yield when the maize forage was pretreated under different severities of dilute acid [16]. It was observed that samples with higher cellulose, reduced lignin and highly substituted hemicelluloses provided significantly higher sugar yields (90 versus 180 g/kg for a combined severity factor of 0.95), but the differences among varieties were reduced by increasing the severity of pretreatment.

The feedstock quality of sugarcane varieties can be improved through classical breeding or precision breeding (genetic engineering), and both of these have shown the possibility to produce sugarcane lines that are less recalcitrant to bioconversion, without affecting plant performance in controlled environmental conditions [13,17]. In this context, the present study evaluated the responses of different sugarcane varieties from classical breeding at various stages of the conversion process: altering pretreatment severity, altering enzyme requirements and the eventual ethanol yield during SSF, and overall ethanol yield considering agronomic data (l ethanol/hectare(ha)) [18]. In a previous study, 115 varieties from the breeding program at the South Africa Sugarcane Research Institute (SASRI) were screened in terms of potential ethanol yields per hectare from both sugar juice and bagasse [6]. Out of the 115 cultivars, the bagasse of three preferred varieties from classical breeding were selected for further optimization together with an industrial bagasse for comparison purposes. In the present study, each bagasse sample was firstly subjected to different pretreatment conditions to determine those that provided the maximum combined sugar yield, while minimizing byproduct formation. This optimization was done by central composite design (CCD) varying the temperature and time for a fixed acid loading. Subsequently the pressed materials pretreated under optimum conditions were used as substrate to carry out SSF with two different enzyme loadings. Additional fed-batch SSF experiments were conducted in order to obtain ethanol concentration of at least 40 g/l. Finally, the overall ethanol yield (l/ha) (biomass yield and ethanol from the juice and bagasse) was also calculated, to identify preferred varieties for bioethanol production.

## Results

### Chemical composition of biomass

The chemical composition of the bagasse samples obtained from classical breeding (55, 70, 74) are summarized in Table 1. The industrial bagasse (120) obtained from the sugar mill was used as reference material. The bagasse samples differed slightly in their chemical composition. The values for glucan, xylan, arabinan, acetyl groups, acid insoluble lignin, ash and extractives ranged from 37.4 to 39.6%, 19.5 to 23.3%, 1.3 to 2%, 2.2 to 3%, 1.3 to 1.9% and 4.3 to 5%, respectively. The sum of all components measured ranged from 91.3% to 93%. This could be attributed to components that were not quantified (such as methyl glucuronic acid) and some degradation of the sugars occurring during the acid hydrolysis [13].

**Table 1:** Chemical compositions of sugarcane bagasse samples

**Component**	**55**	**70**	**74**	**120**
**Carbohydrates**				
Glucan	38.3 ± 0.5	37.4 ± 0.7	38.1 ± 1.0	39.6 ± 0.6
Xylan	23.3 ± 0.4	22.5 ± 0.1	21.6 ± 0.6	19.5 ± 0.3
Arabinan	2.0 ± 0.1	1.7 ± 0.2	1.4 ± 0.2	1.3 ± 0.1
**Lignin**				
Acid soluble	3 ± 0.4	2.9 ± 0.4	2.8 ± 0.4	2.2 ± 0.1
Acid insoluble	17.3 ± 0.4	17.1 ± 0.2	19.5 ± 0.3	20.2 ± 0.2
Acetyl	3.3 ± 0.1	3.0 ± 0.2	2.8 ± 0.1	3.2 ± 0.2
Extractives	4.3 ± 0.9	4.8 ± 1.1	4.3 ± 0.1	5.0 ± 0.5
Ash	1.5 ± 0.1	1.9 ± 0.2	1.2 ± 0.1	1.3 ± 0.3
Mass closure	93.0	91.3	91.7	92.3

Bagasse 55, 70 and 74 are from classical breeding varieties, and 120 is the industrial bagasse. Values are given in % of dry matter.

One way analysis of variance (ANOVA) (data not shown) indicated that xylan and acid insoluble lignin (AIL) were the only components that were significantly different among the various bagasse samples, at a significance level of 0.05 (*P*-values of 0.009 and 0.003 for the xylan and AIL, respectively). The varieties obtained from classical breeding presented significantly higher xylan (21.6 to 23.3%) than the industrial bagasse (19.5%). Regarding the AIL, variety 74 presented values similar to the industrial bagasse (20.2%). Overall, varieties 55 and 70 had the highest amount of total structural carbohydrates (63.6 and 61.6%, respectively) and the lowest lignin content (17.1 to 17.3%). Assuming a conversion of 0.511 g of ethanol/g sugar, the theoretical ethanol yield that could be obtained ranged from 398.2 to 419.8 L/dry ton for the industrial bagasse and variety 55, respectively.

### Dilute H_2_SO_4_ pretreatment and enzymatic hydrolysis

The effect of feedstock properties in pretreatment requirements was evaluated at a range of pretreatment conditions (Table 2). A CCD was applied to evaluate the influence of temperature and residence time on sugars recovery in the different fractions of pretreated material. The impact of pretreatment conditions on the digestibility of the water insoluble solid (WIS) was also studied. Finally, the combined sugar yield (CSY) from the combined pretreatment-hydrolysis process was determined considering sugars solubilization in the pretreatment liquor and sugar released during enzymatic hydrolysis. The pretreatment conditions required to provide the maximum CSY were compared among the different varieties.

**Table 2:** The coded and real value of factors in central composite design

**Substrates**	**Factors**	**Symbols**	**Coded variables**				
			**-1.41421**	**-1**	**0**	**+1**	**+1.41421**
55, 70 and 74	Temperature (°C)	X_1_	175.9	180	190	200	204.1
	Residence time (minutes)	X_2_	2.93	5	10	15	17.07
120	Temperature (°C)	X_1_	180.9	185	195	205	209.1
	Residence time (minutes)	X_2_	1.93	4	9	14	16.07

In order to quantitatively predict the effect of each independent variable on the responses (xylose, glucose and combined sugar yields), regression analysis was performed according to the quadratic model (Equation 2) to fit the responses as function of the experimental conditions. The statistical significance of each factor was determined by ANOVA.

### Effect of pretreatment on sugar recovery and inhibitors formation

The recovery of the main sugars, glucose and xylose in the liquid and solid products from different conditions of dilute acid pretreatment for the bagasse from varieties 55, 70, 74 and 120 included in the present study are listed in Table 3. It is worth noting that the industrial bagasse (variety 120) required more severe conditions of pretreatment to obtain the maximum combined sugar yield. As expected, most of the glucose was retained in the WIS (86 to 97.3%), while xylose was the main component in the pretreatment liquor (contained 53 to 85.3% of the xylose in raw material). Nevertheless, glucose solubilization increased with the severity of pretreatment, reaching a maximum of 11.9% recovered in the pretreated liquor (Run 6, variety 70). This trend was also observed for the industrial bagasse, but the values of glucose in the liquor were lower (2.5 to 7.7%) in spite of the most severe conditions within the CCD. Similarly, xylose recovery in the pretreatment liquor of bagasse 120 was lower (36.4 to 78.6%) when compared to that of varieties 55, 70 and 74 (53 to 85%). However, the xylose recovery in the WIS was higher in 120 (4.1 to 12.6%) for most of the range of pretreatment conditions tested.

**Table 3:** Recovery of glucose and xylose after pretreatment of bagasse samples at different temperature and time

	**Conditions**				**Glucose (%)**								**Xylose (%)**							
	**Temp**	**Time**			**WIS**				**Liquor**				**WIS**				**Liquor**			
**Run**	**(˚C)**	**(min)**	**pH **^**a**^	**log** ***R***^***’***^_***0***_	**55**	**70**	**74**	**120**	**55**	**70**	**74**	**120**	**55**	**70**	**74**	**120**	**55**	**70**	**74**	**120**
1	180(185)	5(4)	2.11(2.20)	0.94(0.91)	95	96	97.3	97	4.9	3.9	2.5	2.5	11	10	11	11	78.5	67	66.8	65.9
2	200(205)	5(4)	2.05(2.11)	1.59(1.58)	91	90	93.9	95	8.2	9.1	5.5	4.6	7.2	5.1	4.5	5.9	71.7	73	74.7	65.7
3	180(185)	15(14)	2.07(2.09)	1.46(1.56)	92	90	94	93	7.3	8.5	5.5	5.7	6.4	8.2	4.1	8.6	79.2	72	65.7	60.6
4	200(205)	15(14)	2.06(2.08)	2.06(2.16)	89	86	88.8	90	10.6	11	8.2	7.7	3.4	5.1	3.7	4.1	61.8	53	54.1	36.4
5	176(181)	10(9)	2.13(2.14)	1.10(1.20)	95	95	96.9	97	3.8	4	2.7	3.2	5.3	15	14	13	66.7	76	69.4	74.4
6	204(209)	10(9)	2.08(2.10)	1.98(2.06)	91	86	89.6	93	7.7	12	8.5	5.4	4.9	4.3	3.7	7.2	62.5	58	59.6	46.3
7	190(195)	3(2)	2.06(2.15)	1.07(0.95)	95	95	96.2	97	4	5.8	3.6	2.7	6.8	7.4	8.2	13	84	74	69.4	62.8
8	190(195)	17(16)	2.06(2.08)	1.82(1.92)	89	89	91.3	94	9.6	11	8.1	7	4.2	7.4	4.9	8.1	67.2	71	67.8	47.5
9	190(195)	10(9)	2.08(2.12)	1.57(1.63)	90	91	92	93	7.7	9.4	6.1	5.5	4.5	7.8	5.3	8.6	75.4	85	82.6	78.6
10	190(195)	10(9)	2.09(2.10)	1.56(1.65)	93	92	94	95	7.3	8.7	6.6	5.2	4.9	7	5.7	7.7	78.2	83	77.3	75.9
11	190(195)	10(9)	2.07(2.11)	1.58(1.64)	93	89	91.6	95	7.5	9.8	6.5	5	4.9	7.4	5.7	7.2	78.4	79	77.1	74

Values are expressed as the percentage of theoretical value (content in raw material). The acid concentration at all pretreatment conditions was kept constant at 0.5% (w/w).

The conditions and values in parenthesis were employed for bagasse 120 to have a better response to pretreatment.

^a^The values showing the pH for varieties 55, 70 and 74 is the average of the three substrates.

WIS, water insoluble solid; Temp, temperature; and *logR*^*’*^_*0*,_ combined severity factor.

**Figure 1:**
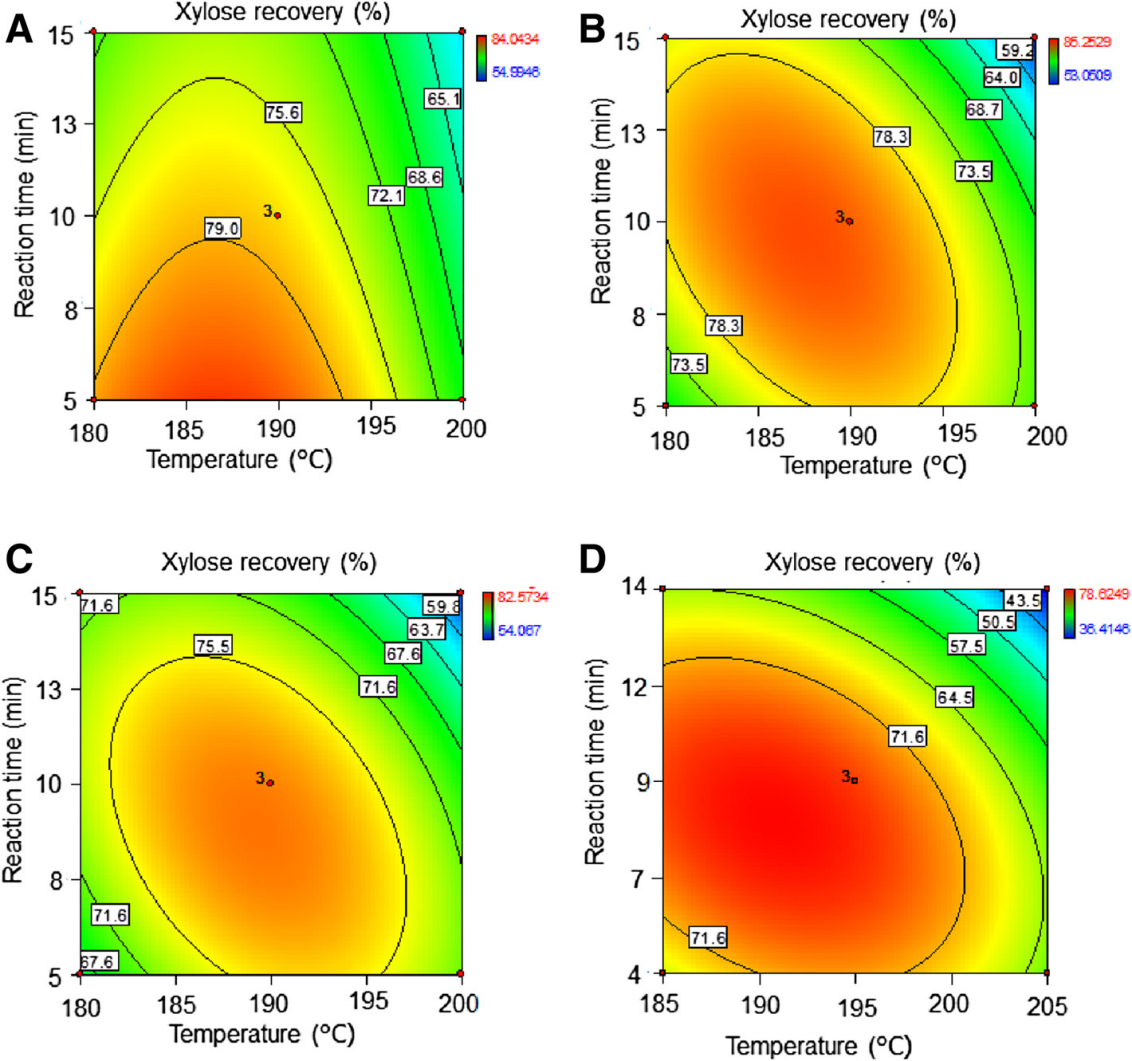
**Contour plots for xylose recovery as a function of pretreatment temperature and reaction time.** The acid loading was kept constant at 0.5% (w/w). The recovery is expressed as percentage of theoretical value for **(A)** variety 55, **(B)** variety 70, **(C)** variety 74, and **(D)** industrial bagasse 120.

Hemicellulose removal from the bagasse fibers is considered as a parameter of the effectiveness of dilute acid pretreatment on accessibility but, depending on its severity, the solubilized sugars can be further degraded into furans [19]. Statistical analysis was used to evaluate the effect of temperature and residence time on recovery of the hemicellulose, specifically xylan in the form of oligomeric and monomeric xylose, in the pretreatment liquor. The equations for the total xylose recovery in the pretreatment liquor for each variety (Table 4, Equations 5-8) were used to draw contour plots (Figure 1). Both temperature and reaction time impacted xylose yield in the pretreatment liquor in a negative manner for all varieties in the range of pretreatment conditions investigated. However, it can also be observed that varieties had different pretreatment requirements. For example, variety 55 required a lower temperature and shorter residence time (186°C for 5 minutes) than the 120 (190°C for 8 minutes) to attain its maximum xylose recovery in the liquor (82.5 and 78.5% for the varieties 55 and 120, respectively).

**Table 4:** Coefficient of determination, optimal conditions and predicted and validated maximum values for different optimization criteria

**Model (in coded form)**		**R**^**2**^	**Optimal conditions**		**Max. values**	
			**Temperature**	**Time**	**Prediction**	**Value**
5	*X*_*55*_ *= 77.15-3.77 T-4.12 t-5.64 T*^*2*^	0.85	186	5	82.5	Na
6	*X*_*70*_ *= 82.33-4.80 T-2.50 t-6.15Tt-8.51 T*^*2*^*-5.94 t*^*2*^	0.91	187	9	82.9	Na
7	*X*_*74*_ *= 78.99-2.20 T-2.99 t-4.88Tt-7.56 T*^*2*^*-5.50 t*^*2*^	0.88	188	9	79.3	Na
8	*X*_*120*_ *= 76.18-8.00 T-7.02 t-6.01Tt-8.06 T*^*2*^*-10.66 t*^*2*^	0.97	190	8	78.5	Na
9	*G*_*55*_ *= 38.05 + 3.63 T + 1.79 t-2.7 3 T*^*2*^*-2.91 t*^*2*^	0.98	193	10	38.7	Na
10	*G*_*70*_ *= 34.99 + 2.73 T + 1.44 t-2.61Tt-2.61 T*^*2*^	0.98	197	6	36.1	Na
11	*G*_*74*_ *= 26.54 + 3.24 T + 2.63 t*	0.88	194	15	30.4	Na
12	*G*_*120*_ *= 27.67 + 2.25 T + 2.85 t-1.39Tt*	0.97	198	14	31	Na
13	*CSY*_*55*_ *= 65.47 + 2.16 T + 1.04 t-4.90 T*^*2*^*-2.91 t*^*2*^	0.96	189	10	65.3	65.8
14	*CSY*_*70*_ *= 63.73 + 1.80 T + 1.26 t-4.55Tt-5.29 T*^*2*^*-1.96 t*^*2*^	0.99	188	11	63.5	64.5
15	*CSY*_*74*_ *= 52.67 + 3.05 T + 2.54 t-2.70Tt-2.85 T*^*2*^*-2.44 t*^*2*^	0.95	189	12	53	52.7
16	*CSY*_*120*_ *= 49.24 + 0.35 T + 1.64 t-2.82Tt-2.19 T*^*2*^*-2.08 t*^*2*^	0.92	192	12	49.7	50.3

*X, G* and *CSY* stands for xylose recovery after pretreatment (% of theoretical), glucose yield after enzymatic hydrolysis (g per 100 g dry material) and combined sugar yield (g per 100 g dry material); subscripts (55, 70, 74 and 120) stands for substrates.

*T* and *t* represents temperature and reaction time in coded form.

Optimal conditions: Temperature (°C) and time (minutes); acid concentration was kept constant at 0.5% (w/w).

Prediction. and Value stands for the maximum values predicted by the model and those obtained experimentally, respectively.

Na means not determined.

R^2^, coefficient of determination.

**Figure 2:**
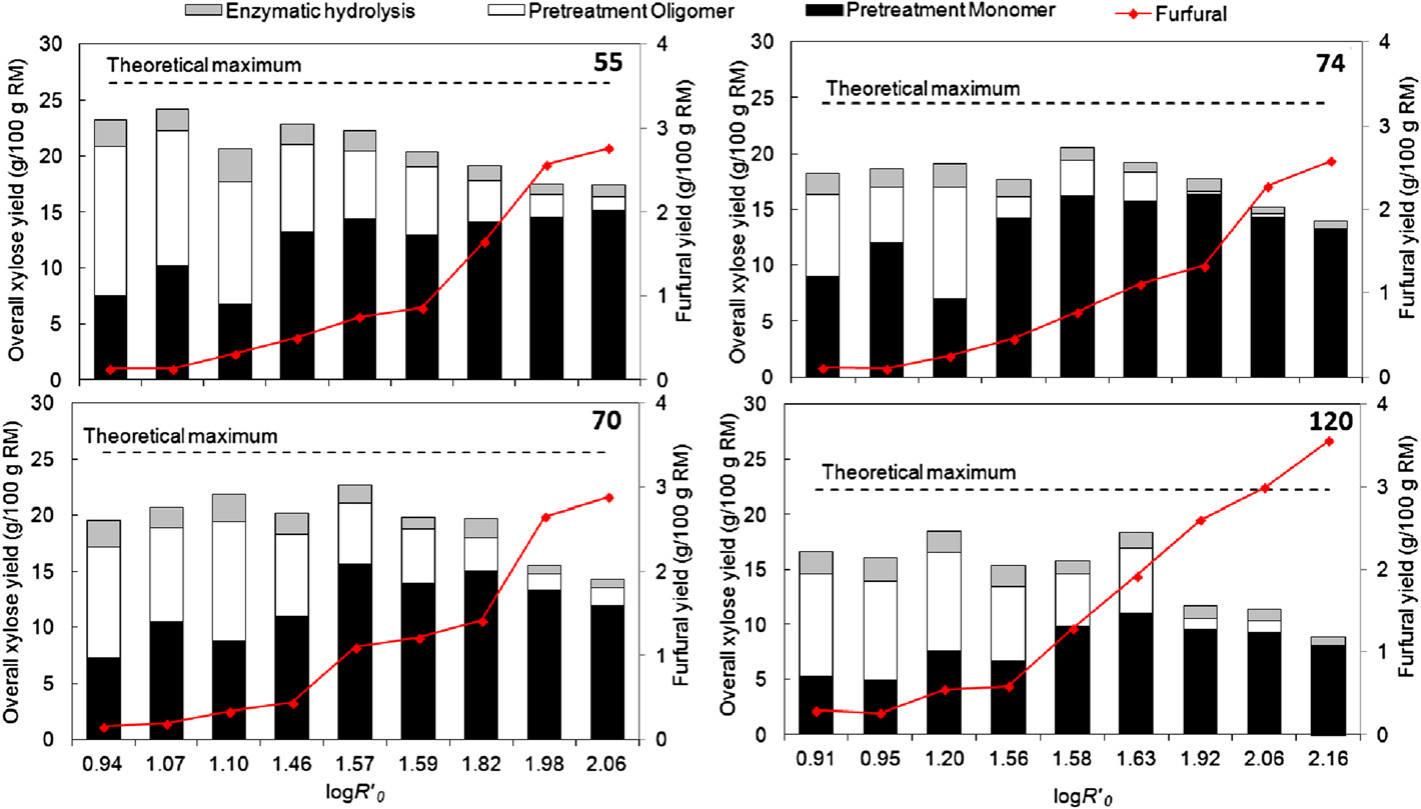
**Overall xylose yield and furfural formation as the function of combined severity factor.** The yield is expressed as gram per 100 g raw material (RM) for varieties 55, 70, 74 and industrial (120). The theoretical maximum for each feedstock is also indicated by discontinuous lines. Varieties 55, 70 and 74 were pretreated with similar conditions.

Another parameter desirable for fermentation processes is the presence of sugars in monomeric form. The amount of xylose recovered in liquid fraction in monomeric or oligomeric (xylo-oligomers, XOS) form is depicted in Figure 2. No XOS were detected in the pretreatment liquor for the most severe pretreatment conditions applied to bagasse from varieties 74 and 120. The maximum XOS yield, about 14 g/100 g raw material (RM), was obtained for the variety 55 for the lowest CSF (0.96), which corresponded with 64% of the total xylose in the liquor. Although higher severities resulted in a higher proportion of xylose in monomeric form, these conditions also increased xylose degradation into byproducts (Figure 2). Up to 59.5% of theoretical xylose was degraded (variety 120, CSF of 2.16). Nonetheless, the levels for furfural (3.6 g/100 g RM) and formic acid (0.2 g/100 g RM), degradation product from xylose and furfural respectively [20], did not account for all the xylose lost, similar to previous reports on optimization of dilute acid pretreatment [21].

Regarding the total amount of inhibitors present in the pretreatment liquor, similar values were observed for the four substrates evaluated. Acetic acid, originated through hydrolysis of the acetyl groups of the hemicelluloses [22], was the inhibitor present in the liquor at the highest concentration. The acetyl hydrolysis into acetic acid increased with severity of the pretreatment, and it maxed out at CSF of 1.98 and 2.16 for classical breeding varieties and industrial bagasse, respectively (data not shown). Under these conditions, the acetyl group hydrolyzed was more than 96 to 98.4% of theoretical. Another difference observed among varieties was the presence of hydroxymethylfurfural (HMF) at concentrations ranging from 0.091 to 0.782 g/100 g RM in the pretreatment liquors from the varieties 55, 70 and 74, while for the industrial bagasse the highest concentration was 0.218 g/100 g RM (data not shown).

### Effect of pretreatment on enzymatic hydrolysis of washed pretreated solids

As mentioned earlier, the undesired feedstocks properties, present in differential of the same feedstock, can require increasing severity in process requirements (pretreatment and enzymatic hydrolysis). In an attempt to study the effect of feedstock-pretreatment combination on enzyme susceptibility, the WIS fraction of each variety-pretreatment combination was subjected to enzymatic saccharification. Enzymatic hydrolysis of the untreated materials was included for comparison.

The cellulose conversion of untreated materials was less than 30%. The differences in the recalcitrance of the bagasse of the different varieties could already be observed on the enzymatic hydrolysis of the untreated material. The untreated bagasse from varieties 55 and 74 provided between 6 and 9% greater cellulose conversion than that of the variety 74 and industrial bagasse. Dilute acid pretreatment considerably increased the glucan conversion compared to untreated bagasse, giving values from between 48.6 and 66.4% for the less severe conditions, to 100% for the harshest conditions (Table 5). The differences in digestibility observed between varieties 55 to 70 and 74 to 120 were more evident after applying the pretreatment. The varieties 55 to 70 seemed to be less recalcitrant, requiring severities of about 1.6 to reach a digestibility higher than 80%, while the bagasse from variety 74 and industry (120) needed severities of at least 1.9 to reach digestibilities close to 80%.

**Table 5:** Glucose yield/recovery and combined sugar yield after pretreatment and enzymatic hydrolysis of bagasse samples

**Run**	**Enzymatic hydrolysis**								^**b**^**Overall glucose recovery (%)**				^**c**^**Combined sugar yield (g/100 g RM)**			
	**Glucose yield (g/100 g RM)**				^**a**^**Digestibility (%)**											
	**55**	**70**	**74**	**120**	**55**	**70**	**74**	**120**	**55**	**70**	**74**	**120**	**55**	**70**	**74**	**120**
Untreated	11.7	12	9.2	8.9	27.5	28.6	21.7	20.2	-	-	-	-	15.7	17	13	12.5
1	26.7	25	21	20.8	66.4	63.6	50.5	48.6	68.1	65.0	56.7	50.0	54.3	48	40.6	39.6
2	33.9	36	29	29	87.6	97.0	71.9	69.3	86.9	96.3	73.2	70.5	59.6	62	51.8	47.6
3	30.6	34	27	29.6	78.6	91.6	68.4	72.3	79.9	91.4	70.9	72.7	58.6	60	48.8	48.3
4	36.5	35	31	32.2	96.6	97.1	81.1	81.1	96.3	96.3	80.3	81.8	60.3	55	49.3	45
5	27.4	26	20	24.5	67.5	64.8	49.7	57.5	68.1	65.0	52.0	59.1	51.5	51	42.2	45.3
6	38.7	33	31	29.6	100.0	92.9	81.5	72.6	98.7	91.4	82.7	72.7	58.7	55	51.2	44
7	29.9	33	21	24.3	74.3	84.5	50.8	56.8	75.2	86.6	52.0	59.1	58	58	42.4	42.4
8	37.5	36	30	31.9	99.2	97.2	76.8	77.1	98.7	96.3	78.0	79.5	62.3	62	52.7	47.4
9	38	35	28	27.7	99.3	92.4	70.9	67.4	96.3	93.9	70.9	68.2	64.8	65	53.3	50.3
10	37.8	35	28	27.4	95.4	92.8	70.1	65.8	96.3	93.9	73.2	68.2	65.5	64	52.6	49.1
11	38.4	35	28	27.3	97.4	94.4	71.2	65.6	98.7	93.9	70.9	68.2	66.1	63	52.1	48.3

The acid concentration at all pretreatment conditions was kept constant at 0.5% (w/w).

^a^Digestibility was calculated as glucose yield divided by the potential glucose in the WIS expressed in percentage.

^b^Overall glucose recovery is the sum of glucose obtained after pretreatment and enzymatic hydrolysis divided by potential glucose in the native material expressed in percentage.

^c^Combined sugar yield is the sum of glucose, xylose and arabinose obtained after pretreatment and enzymatic hydrolysis.

RM, raw material; WIS, water insoluble solid.

**Figure 3:**
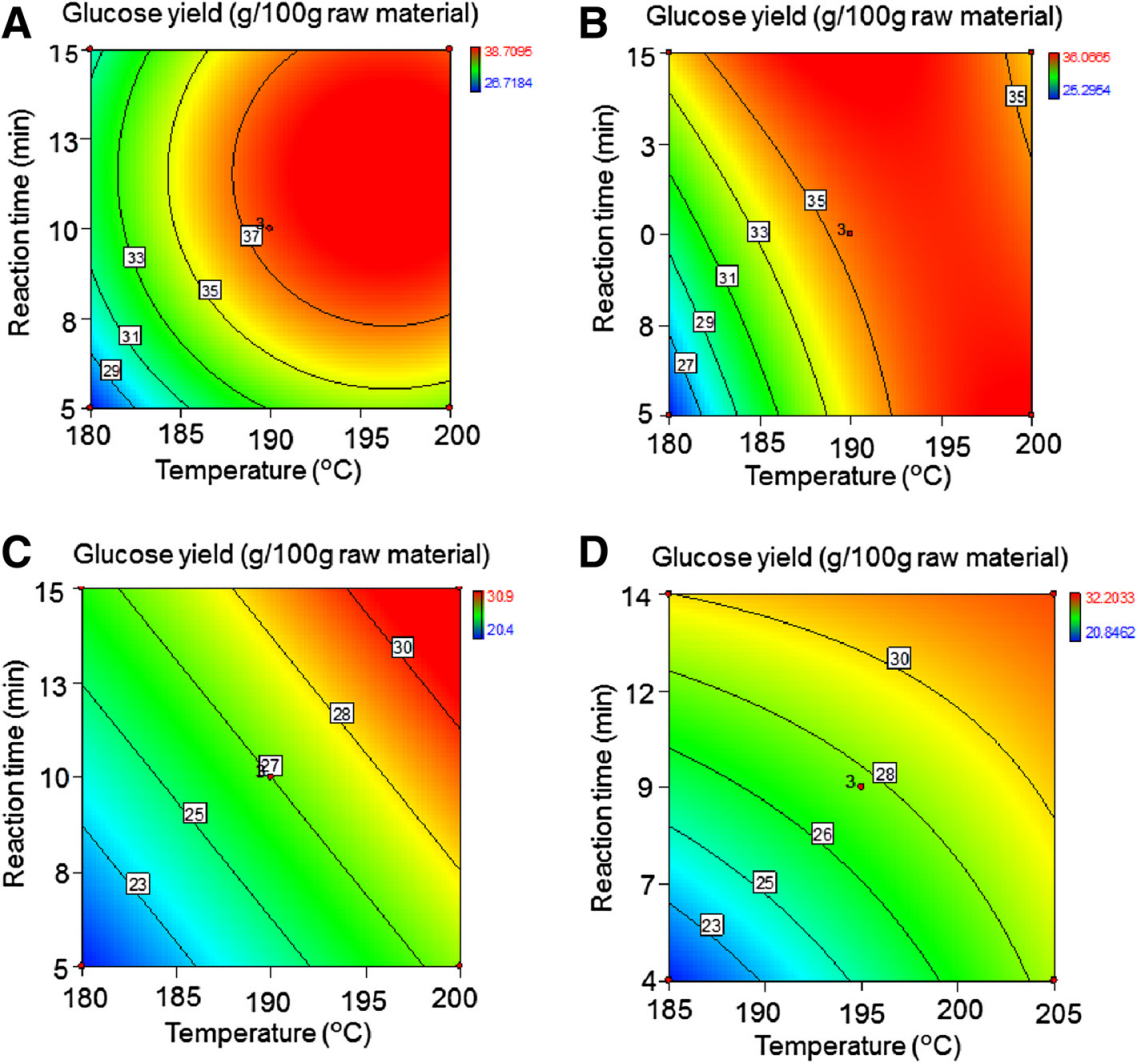
**Contour plots for glucose yield after enzymatic hydrolysis as the function of pretreatment temperature and time.** The acid concentration was kept constant at 0.5% (w/w). The yields are expressed as grams per 100 grams of dry bagasse for **(A)** variety 55, **(B)** variety 70, **(C)** variety 74, and **(D)** industrial 120.

Although more severe pretreatment conditions generally improve the accessibility of the fibers during enzymatic hydrolysis, it is normally at the expense of xylose degradation and lower fiber recovery in the pretreatment. The glucose yield from the fibers, considering the insoluble solids recovery of the pretreatment and the glucose released during enzymatic hydrolysis, was evaluated statistically. The effect of pretreatment conditions on glucose yield (g/100 g RM) is represented in contour plots (Table 5, Equations 9-12) in Figure 3. As expected, the highest yields of glucose were obtained at higher temperatures than those for maximum xylose yield (Figure 1). It was also found that the glucose yield for variety 74 could only be determined by the linear effects of temperature and residence time. Similarly to what was observed for xylose yields, the varieties 55 and 70 required less severe conditions to reach the maximum glucose yield when compared to the other varieties.

### Combined sugar yield

The sugars solubilized in the pretreatment liquor, together with those released during enzymatic hydrolysis subsequent to pretreatment, were used to determine the CSY (Table 5). The range of pretreatment conditions evaluated gave 9 to 15% differences in the CSY between the less and more harsh pretreatment conditions. The highest value of CSY was obtained for the central point for all the varieties evaluated (190°C for 10 minutes for the varieties 55, 70 and 74; 195°C for 9 minutes for industrial bagasse). The highest CSY were obtained for varieties 55 and 70 with respective average values of 65.5 and 63.7 g per 100 g dry material, respectively, which corresponds with 91.9 and 92.2% of sugars present in the raw material, respectively. Interestingly, the conditions that gave the highest CSY also generated a lower concentration of inhibitors than those that gave the highest glucose yield (Figure 3). The concentrations of furfural (0.5 to 1 g/l), HMF (0.1 to 0.3 g/l), acetic acid (1 to 1.5 g/l) and formic acid (0.04-0.06 g/l) determined at these conditions (for the highest CSY) were under the threshold toxicity for *Saccharomyces cerevisiae* reported previously (0.3, 1.2, 2 to 6, 0.8 g/l for HMF, furfural, acetic acid and formic acid, respectively) [23,24].

The mathematical models for combined sugar yield containing the significant terms in coded form are also summarized in Table 4 (Equations 13-16). The statistical significance of each model was determined by ANOVA, which revealed that the models were significant, while the lack of fit was insignificant (*P* < 0.05) (not shown). The models were further validated by performing additional experiments at the optimum conditions identified by the model (Table 4). The experimental CSY values differed less than 2% from the predicted values. These conditions were therefore selected to generate substrate for SSF experiments.

**Figure 4:**
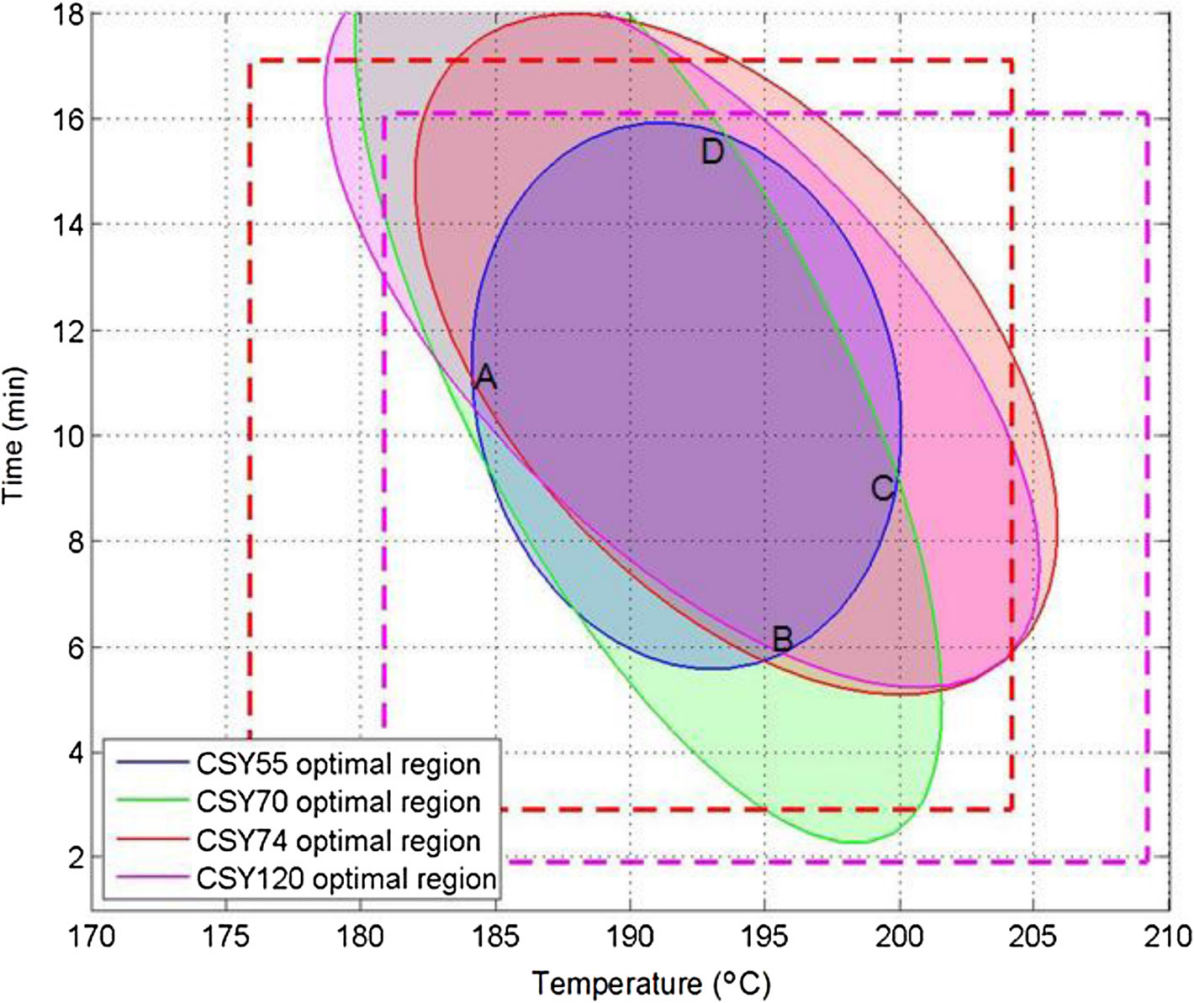
**Contour plots showing the pretreatment conditions that provide 95% of the maximum combined sugar yield.** The yield is expressed as gram per 100 g dry bagasse. The dotted lines represent the input range of the independent variables for the cultivars: red for varieties 55, 70 and 74; pink for bagasse 120. Area **A, B, C and D** is the intersection area that shows a common range of pretreatment conditions that provide 95% of the maximum combined sugar yield for all varieties. CSY, combined sugar yield.

The maximum CSY values varied for the different varieties, but there was a common range of pretreatment conditions providing the maximum CSY for all varieties. Equations 13-16 (Table 4) were used in the Matlab program, version 8.1 (R2013a) (MathWorks, Masshachuttes, United States) in order to represent the pretreatment conditions that will reach 95% of the maximum combined sugar yield for each of the bagasses (Figure 4). It could be observed that variety 55 had a narrower set of conditions, giving the maximum CSY followed by variety 70, 120 and 74. The area in common for the four samples evaluated is observed in the range of conditions defined by the intersection region (ABCD), which were between 184 and 200°C for temperature and varying residence time to give a severity factor of between 3.51 and 3.96.

### Effect of pretreatment on SSF of unwashed pretreated solids

The batch SSF was performed on the unwashed pressed slurry from optimum pretreatment conditions for maximum CSY, at a solids loading of 10%. The slurry was pressed up to a final moisture content of between 59 and 63%. The use of the pressed slurry presents some advantages for the process such as the avoidance of both the washing step and loss of sugars. Some of the sugars remain soaked in the fibers, providing extra fermentable sugars for fermentation.

**Figure 5:**
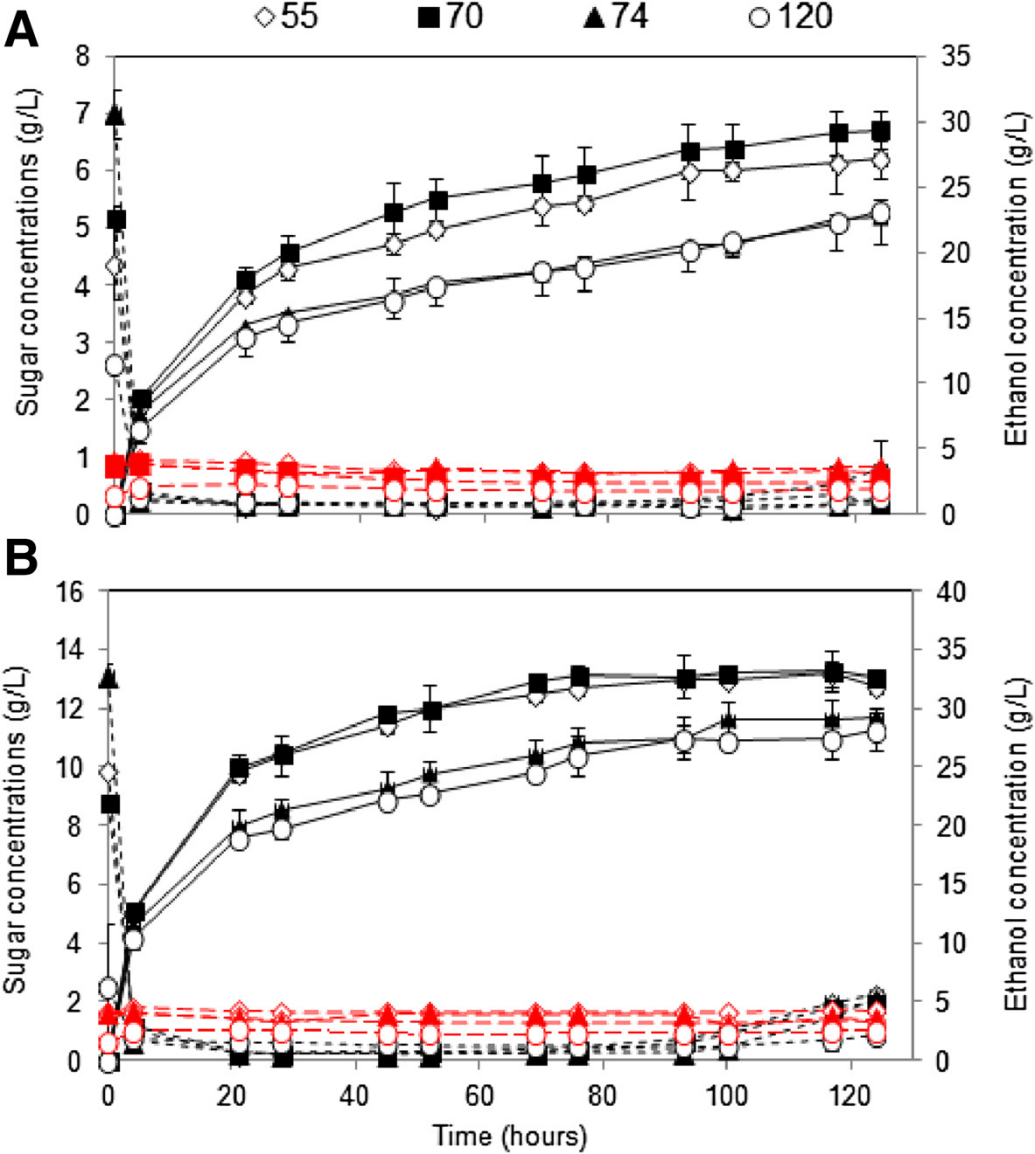
**Glucose, xylose and ethanol concentrations (g/L) during batch SSF of dilute acid pretreated bagasse samples.** Glucose (in dotted lines in black), xylose (dashed lines in red) and ethanol (in solid lines in black). Conditions: solid loading 10% (w/v), **(A)** 0.15 mL of Cellic Ctec2/g WIS and 0.0167 mL of Cellic Htec2/g WIS **(B)** 0.15 mL of Cellic Ctec2/g WIS and 0.213 mL of Cellic Htec2/g WIS. Samples were pretreated based on optimum conditions for maximum combined sugar yield. 55, 70, 74, 120, varieties of sugarcane; WIS, water insoluble solid; w/v, weight by volume.

The time course for glucose, xylose and ethanol concentrations during batch SSF of dilute acid treated samples for the two enzyme dosages at a solids loading of 10% are illustrated in Figure 5. All four substrates showed similar profiles. The initial glucose (from the pretreatment liquor and the glucose generated during the prehydrolysis) was rapidly consumed within the first 8 hours and remained at values close to zero until the end of SSF in the case of low enzyme dosage (Figure 5A), or until 100 hours when Htec2 was manually increased, and Ctec2 held constant (Figure 5B). As a result, ethanol concentrations were gradually increasing until these periods. The xylose, however, remained constant for the entire process.

The highest final ethanol concentrations were attained when using the varieties 55 and 70 for both enzyme dosages (Figure 5). No significant differences in ethanol yields were observed between variety 55 and 70, and between variety 74 and bagasse 120. The highest ethanol concentrations at low enzymes loading (0.15 ml of Cellic Ctec2/g pretreated material and 0.0167 ml of Cellic Htec2/g pretreated material) were 27.1, 29.3, 22.8 and 23.1 g/l for varieties 55, 70, 74 and bagasse 120, respectively (Figure 5A). These corresponded to ethanol yields of 69.5%, 75.8%, 62.5% and 61.2% of theoretical maximum, based on glucose content in the pressed slurry. Ethanol productivity (the highest ethanol concentration divided by the total time taken to the maximum concentration) were 0.219, 0.236, 0.184 g^-1^ h^-1^ and 0.186 for varieties 55, 70, 74 and 120, respectively. As expected, increasing the enzyme dosage resulted in higher ethanol concentrations. Ethanol concentrations of 33.0, 33.1, 29.1 and 28.1 g/l, corresponding with ethanol yields of 84.5%, 85.6%, 79.9%, and 74.8% of the maximum theoretical yield based on glucose content in pressed material for varieties 55, 70, 74 and bagasse 120, respectively, were obtained (Figure 5B). Ethanol productivity could be increased from 0.184 - 0.236 g^-1^ h^-1^ to 0.227 - 0.283 g^-1^ h^-1^ by applying high enzyme dosage. It is worth noting that a higher enzyme dosage was required for varieties 74 and 120 in order to obtain similar ethanol concentrations to those obtained at the lower enzyme dosage for varieties 55 and 70.

**Figure 6:**
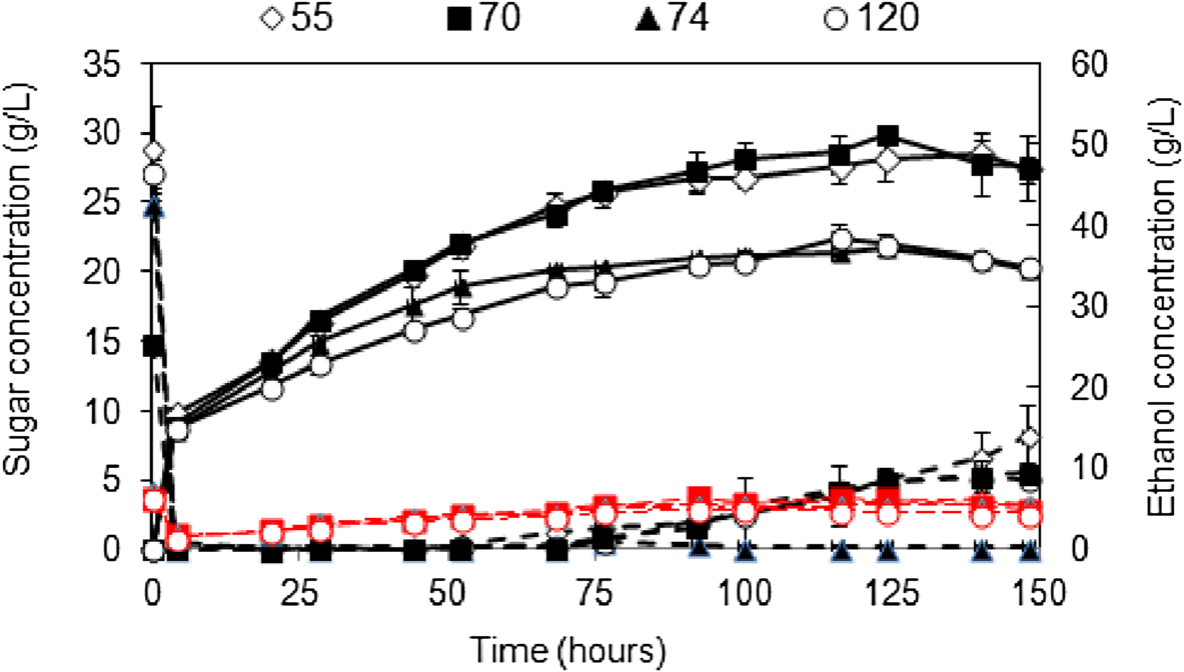
**Glucose xylose and ethanol concentrations (g/L) during fed-batch SSF of dilute acid pretreated bagasse samples.** Glucose (in dotted lines in black), xylose (dashed lines in red) and ethanol (in solid lines). Solid loading (16% (w/w)) and at enzyme dosage (0.15 mL of Cellic Ctec2/g WIS and 0.213 mL of Cellic Htec2/g WIS). Samples were pretreated based on optimum conditions for maximum combined sugar yield.

In an attempt to reach at least 40 g/l of ethanol [25], a fed-batch strategy was adopted for SSF to increase the dry matter concentration to 16% (w/w), while avoiding mass transfer limitations. Figure 6 depicts concentrations of xylose, glucose and ethanol for all four substrates during the fed-batch SSF. Similarly to batch SSF, the ethanol concentration progressively increased while the residual glucose concentration remained at almost zero for the first 76 hours of SSF. However, glucose levels at the beginning of the SSF were higher than those were anticipated. This suggests that the addition glucose came from Ctec2/Htec2 enzymes (personal communication: A Rudolph, Novozymes, Denmark). However, this does not affect the differences in ethanol yields among varieties. The level of xylose slightly increased during SSF probably due to the residual xylose in liquor soaked in the fibers and/or the xylan-degrading enzyme present in cocktails combinations. Ethanol concentrations higher than 40 g/l were reached only for varieties 55 and 70 after 68 hours of SSF. The highest ethanol concentrations were 51.3 and 48.6 g/l, which correspond to ethanol yields of 78.4 and 74% of theoretical maximum based on glucose in pressed slurry for varieties 70 and 55, respectively. For varieties 74 and industrial bagasse 120, the highest ethanol concentrations were 37.1 and 38.3 g/l, which were equivalent to 60.6% and 60.1% of the theoretical maximum based on glucose in the presses material. Ethanol productivity for varieties 55 and 70 were also higher (0.348 to 0.414 g^-1^ h^-1^) than that for variety 74 and industrial bagasse 120 (0.299 to 0.331 g^-1^ h^-1^).

**Figure 7:**
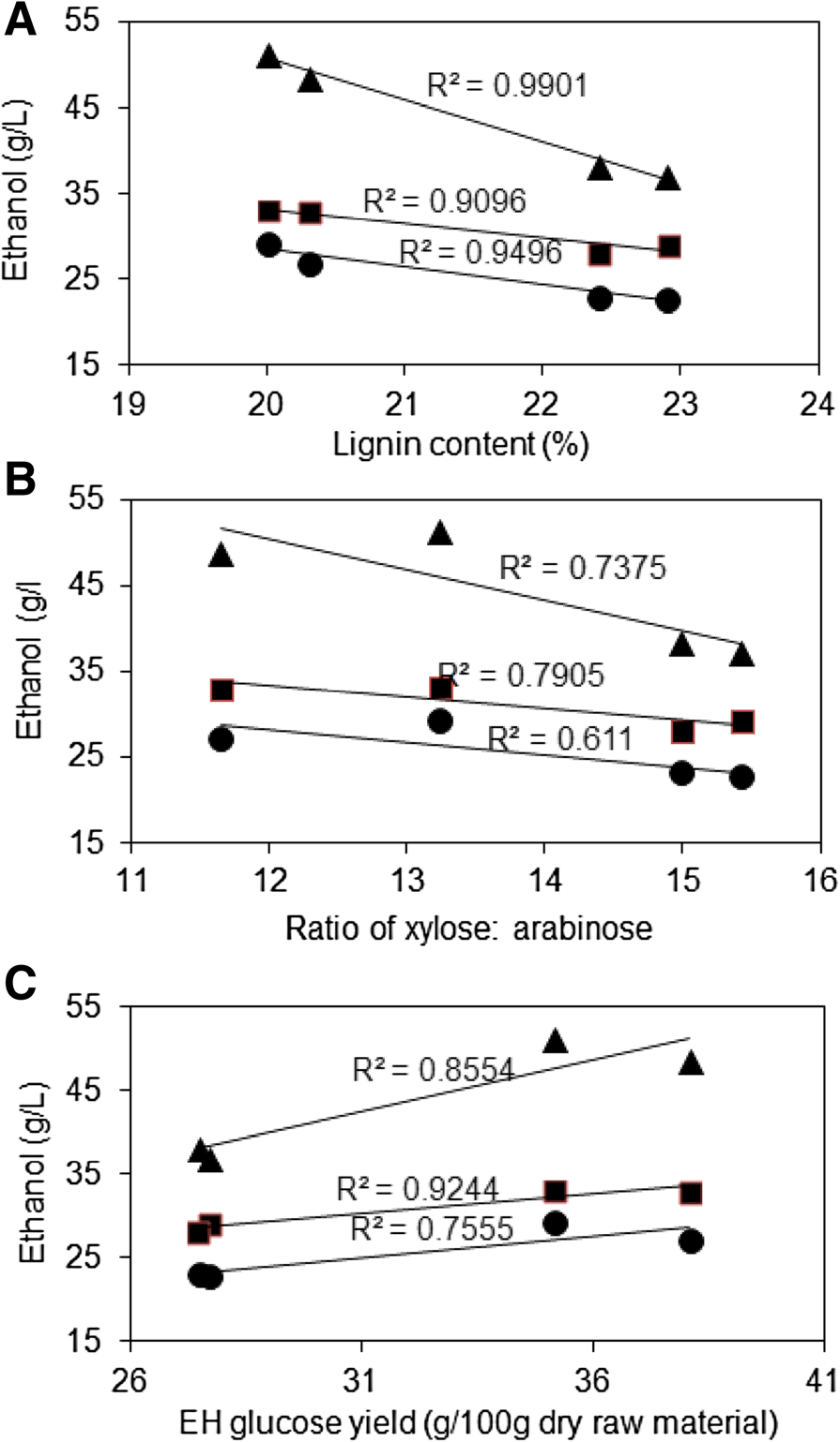
**Correlations between the highest ethanol concentration and lignin, ratio of xylose: arabinose, glucose yield. (A)** Relationship between lignin and ethanol concentration, **(B)** Relationship between the ratio of xylose to arabinose and ethanol concentration, **(C)** Relationship between glucose yield after enzymatic hydrolysis and ethanol concentration. Circle and rectangle markers represent ethanol concentration at low and high enzymes loadings for batch process, whereas triangular markers show the ethanol concentration during fed-batch process.

There were correlations between lignin, xylose: arabinose ratio and glucose yield with ethanol yield. The impact of lignin content and ratio of xylose: arabinose on the ethanol concentration/yield was estimated by calculating coefficient of determination between them. In addition, relationship between the glucose yield and ethanol concentration was also established. The correlation was based on the highest ethanol concentration obtained by each substrate as depicted in Figure 7. As for the case of glucose yield (Table 5), most of the variation in ethanol concentration/yield was largely attributed to differences in lignin content between the varieties. Strong inverse correlation (R^2^ = 0.9098 to 0.9901) between lignin content and ethanol concentration was observed (Figure 7A). The study observed inverse correlations (R^2^ = 0.611 to 0.7375) between ethanol concentration and the ratio of xylose: arabinose (Figure 7B) but, as expected, there was strong positive correlation with the ethanol concentration and glucose yield (R^2^ = 0.7555 to 0.9244) (Figure 7C).

### Estimation of combined ethanol yield

The integration of second generation ethanol production from the bagasse into a first generation ethanol production from sugarcane juice is considered a feasible strategy for industrial implementation, due to the potential for the integration of process unit operations such as feedstock handling, fermentation, distillation and energy utilities (steam, electricity). Moreover, the economics of global ethanol production is highly influenced by agronomic properties of the cultivars such as biomass and juice yield. In this context, the combined ethanol yield for each variety of sugarcane was estimated considering agronomic data, ethanol production from the sugar juice (based on Equation 4) and ethanol production from the bagasse based on the results obtained in this study.

Table 6 summarizes the agronomic properties and values for ethanol yields for the different varieties of sugarcane adopted from previous study (unpublished data submitted for publication: Y Benjamin, J Görgens and S Josh). The average cane productivity, content of soluble sugars in the juice and fiber content for the industrial sugarcane were obtained from literature and were assumed to be 65 wet ton/hectare, 0.13 g/g cane and 0.13 g/g cane, respectively [26]. Conversion efficiency of the sugar from the juice of all varieties in the present study was assumed to be 85% [27], and the ethanol yield was calculated on the basis of cane yield and sugar content on the juice (Table 5). The ethanol yield obtained from bagasse from classical breeding varieties, was calculated based on bagasse yield per hectare (Table 6), and the conversion efficiency was obtained experimentally (highest ethanol yield obtained under the fed-batch SSF, 57.3 to 77%). Additionally, the potential ethanol yield from bagasse was calculated, taking into account the extra ethanol that could be produced if the xylose (pretreatment liquor) was also fermented, assuming a conversion efficiency of 0.18 g ethanol/g xylose consumed [28].

**Table 6:** Ethanol yield per hectare from different varieties of sugarcane (55, 70, 74 and 120)

	**55**	**70**	**74**	**120**
Cane yields (wet ton/ha)	105.5^a^	108^a^	105.5^a^	65^b^
Sucrose content (kg/ton wet cane)	127.5 ^a^	152 ^a^	137 ^a^	126^b^
Sugar juice content (kg/ton wet cane)	149^a^	163^a^	149^a^	140^b^
Bagasse content (kg/ton wet cane)	147^a^	127^a^	161^a^	133^b^
Ethanol yield (L/ha)				
^1^Juice ethanol	9 045	10 169	9 074	5 248
^2^Bagasse ethanol (glucose + xylose)	3 888	3 554	3 574	1 814
^3^Combined (juice + bagasse)	12 933	13 723	12 648	7 062

^a^The cane yield, sugar juice, sucrose and bagasse content for varieties 55, 70 and 74 were provided by SASRI, and they represent the average of two harvests (2009 and 2011).

^b^The cane yield, sugar juice and bagasse content for sample 120 were obtained from the literature according to the average values of the industrial sugarcane.

^1^Juice ethanol is the ethanol that can be produced by fermentation of juice sugars (sucrose, glucose and fructose).

^2^Bagasse ethanol is the ethanol that can be produced by fermentation of the xylose obtained after pretreatment and SSF of the pretreated bagasse.

^3^Combined ethanol yield is the realistic total ethanol that can be obtained from the sugar juice and bagasse.

ha, hectare.

Differences in the combined ethanol yields were observed among the varieties, with varieties from classical breeding (55, 70 and 74) being superior to industrial sugarcane. Variety 70 showed higher combined ethanol yield (13,723 l/ha), whereas variety 55 was superior in terms of ethanol from the bagasse (3,888 l/ha). Juice ethanol yields ranged 5,248 to 10,169 l/ha. The ethanol yields could be increased by 35 to 43% by combining first and second generation technology. Ethanol yield from the fermentation of cellulose was 1,481 to 3,163 g/ha, and could be increased to 1,814 to 3,888 g/ha if the xylose recovered in the pretreatment liquor (at optimal conditions for maximum CSY) was also fermented.

## Discussion

The combination of cultivar selection and process optimization have the potential to enhance sugar conversion efficiency and increase ethanol output per feedstock and further reduce pretreatment severity and enzyme requirement, thereby reducing operating cost [13,14,29].

The bagasse from varieties 55 and 70 presented better response to pretreatment in terms of xylose recovery (Table 3), enzyme digestibility (Table 5) and therefore, combined sugar yield (Table 5). Likewise, these varieties had the highest ethanol yield (conversion efficiency) and final concentrations. This superior performance could be attributed to differences in chemical composition and structure between the samples (Table 1 and Figure 7). For example, varieties from classical breeding (55, 70 and 74) presented with a higher xylan content than industrial bagasse, but no strong correlation between xylan content and xylose recovery was determined for many instances (data not shown). Whereas some other studies on herbaceous biomass also indicated insignificant correlation between xylose recovery and xylan content (transgenic switchgrass and alfalfa [30] as well as forage sorghum [12]), other studies revealed lowest xylose recovery for those varieties with higher xylan content (silvergrass [31]). These weak correlations could be related to xylose degradation during pretreatment and/or differences in the xylan structure. In fact, the feedstocks evaluated in this study could be clustered in the pairs 55-70 and 74-120 according to the degree of arabinose substitution of the xylan backbone (ratio xylose: arabinose of 11.7 to 13.2 and 15 to 15.5 for the varieties 55-70 and 74-120, respectively).

In terms of glucose yield, higher digestibility of the pair 55-70 was also obtained even when no pretreatment was applied. This could be attributed to the lower lignin content compared to the pair 74-120. It is well known that the lignin matrix inhibits the cellulases, acting not only as a structural barrier, but also by the unproductive binding of enzymes, thus leading to lower cellulose digestibility [32]. Moreover, the digestibility obtained after the different pretreatment conditions presented a negative correlation with the ratio xylose: arabinose (not shown). It has been hypothesized that hemicelluloses with lower degrees of substitution are more likely to re-bond to the cellulose during mild dilute acid pretreatments [16]. This point is further supported by the higher recovery of xylan in the WIS from industrial bagasse compared to the classical breeding varieties (Table 3) for most of the pretreatment conditions evaluated.

However, the differences in cellulose digestibility between the best and worst performing varieties did not decrease when increasing the severity. This observation differs from the results found in previous studies when the pretreatment was conducted in a tubular reactor [12,16,29]. This could be due to the fact that the range of pretreatment conditions evaluated was close to the optimum for CSY.

As indicated in Table 4 and Table 5, the pair 55-70 provided the greater CSY at less severe pretreatment conditions than the industrial bagasse. Moreover, the conditions for the maximum CSY did not lead to substantial sugar degradation when compared to those conditions that gave the highest glucan conversion (Table 4). This observation suggests that the conditions for the highest CSY is of more benefit to ethanol production than maximizing glucose yield, as more than 90% of theoretical sugar was recovered with the best performing varieties. The preferred varieties pretreated under optimum conditions provided up to 33% increment of CSY compared to industrial bagasse. Nevertheless, despite the different pretreatment requirements between varieties (Figures 1, 3, 4; Tables 4 and 5), the bagasse from the 4 varieties presented a range of conditions in common (temperature between 184 and 200°C and varying residence time to give the severity factor between 3.51 and 3.96) where the maximum CSY could be obtained (Figure 4). Although further research is needed for confirmation, this finding can constitute a promising tool to select optimum conditions without the pretreatment optimization according to variety.

As expected, SSF process with the pair 55-70 resulted in higher ethanol concentration and ethanol yield for all the SSF processes evaluated. However, a fed-batch strategy was required in order to reach more than 40 g/l. Moreover, it appears that these feedstocks had less enzyme requirement, probably due to the lower lignin content and higher branched xylan (Table 1 and Figure 7). Interestingly, the preferred varieties (55 and 70) in terms of sugars and ethanol yields efficiency (Table 4, Figures 5 and 6) also showed higher combined ethanol per unit hectare compared to the industrial sugarcane (Table 6). The projected combined ethanol yield for these preferred varieties of ‘energycane’ was almost twice of that observed for industrial sugarcane. These results suggest that ethanol yield per hectare can be improved through crop development and integrated conversion approach (‘whole plant’, use of pressed slurry, SSF).

## Conclusions

The present study provides evidence of the impact of cultivar selection and process optimization in sugar conversion efficiency and ethanol output per feedstock. Experimental results show that varieties with reduced lignin content and highly substituted xylan resulted in higher sugar and ethanol yields with milder pretreatment conditions and reduced enzyme dosage, which in turn could reduce the operating cost without causing detriment to the ethanol production from the juice.

## Materials and methods

### Raw material and sample preparation

The sugarcane varieties were developed by the South African Sugarcane Research Institute (SASRI) through classical breeding towards a higher biomass yield [6]. The experimental field trial was conducted at SASRI, Mount Edgecombe, KwaZulu Natal, South Africa (latitude: 29.7000° S; longitude: 31.0333° E). The genotypes were first planted in the field in 2006. The genotypes used in this study were from the fifth ratoon. The plants were rain fed and no fertilizer was used. The genotypes were 99 F2004^55^, 00 F0884^70^ and 01G1662^74^ and all of them had a South African origin. The superscripts (55, 70 and 74) were used for varieties identification. The industrial sugarcane bagasse (labeled 120) was provided by TSB Sugar Mill in Malelane, Mpumalanga, South Africa.

To obtain the bagasse, 20 to 30 of cane stalks (not less than 6 kg) per clone per plot were randomly cut from the experimental field in September 2011 (8-month old plants). The stacks were shredded using a mechanical shredder/disintegrator (locally manufactured, Mechanical Department, SASRI, KwaZulu Natal) and then blended with water (1.5 kg of sample and 3 liters of water) for twenty minutes using a mixer (locally manufactured, Mechanical Department, SASRI, KwaZulu-Natal). Thereafter, the finely crushed shredded canes from the blending jar were washed with water (400 g of sample and 1 liter of water) three times and each wash was collected and measured for residue sucrose and other soluble sugars. In the case of industrial bagasse, the sample was washed three times (200 g and 500 ml of water) and each wash was analyzed for residual sugar content.

The remaining fibers after washing were pressed to reduce water content and dried at 40°C for four days until reach a moisture content of 6%. The samples, mixed and sieved in a vibratory sieve shaker model AS200 basic (Resch GmbH, Düsseldorf, Germany) to obtain a representative particle size suitable for the composition analysis and for the pretreatment studies. The particles retained between 600 and 1000 μm were used for composition analysis and for the pretreatment. The samples were quarter sampled and then packed in zipped plastic bags and stored in a temperature and moisture controlled room until needed.

### Dilute acid pretreatment

Dilute acid pretreatment was conducted in 1000 ml Hastelloy C276 Parr reactor with a magnetic driven turbine agitator (Model 4540, Parr Instrument Company, Moline, Illinois, United States). The surface and internal temperatures of reactor were monitored with two thermocouples type Pt. RTD class B (Omega Moline, Parr Instrumentation company) connected controller (Model 4848B, Parr Instrument Company, Moline, Illinois). The vessel was loaded with 60 g (dry weight) and 600 ml of sulfuric acid solution (0.5%w/w), sealed and stirred at 250 rpm via a 4848B controller. The vessel was heated using 4 kW fluidized sand bath (Model SBL-2D, Techne Co., Minneapolis, United States) coupled with a temperature controller (Model TC-8D, Techne, Minneapolis, United States), previously heated to 350°C. The reaction time of pretreatment was initiated once the target temperature was reached. At the end of the reaction time the vessel was quenched by submerging it into cold water. When the temperature of 100°C was reached (within 4 minutes), the vessel was opened.

The pretreated material (slurry) was characterized in terms of total solids, water soluble solids, water insoluble solids and pH [33]. For analytical purposes, the slurry was vacuum-filtered into solid and liquid fractions. The solid fraction was further washed three times with deionized water, 300 ml for each wash. The remaining solids, referred to as water insoluble solid (WIS), were weighed to calculate the insoluble solid recovery. The chemical composition of the WIS was determined as described previously. Likewise, the pretreatment liquor and wash liquor were analyzed for oligomeric and monomeric sugars, sugar degradation products (furfural and HMF), acetic acid and formic acid.

### Experimental design and optimization

A CCD under response surface methodology (RSM) was used in the optimization of dilute acid pretreatment conditions to improve sugar yield from sugarcane bagasse. Temperature and residence time were selected as independent variables. Xylose yield after pretreatment, glucose yield after enzymatic hydrolysis and CSY were considered as the response variables (dependent output variables).

A two level, two factors full factorial design with four axial points, three replicates at centre point leading to total number of eleven experiments, were employed for pretreatment and enzymatic hydrolysis of the bagasse samples. The coded values for the axial, factorial and centre points were (-1.41421 at the lowest point and +1.41421 at the highest point), (-1 and +1), and (0), respectively as shown in Table 2. The uncoded values were calculated according to Equation 1. The selection of the pretreatment conditions were based on previous studies [29]. The acid loading and solids loading were kept constant at 0.5% (w/w) and 10% (w/v), respectively. The complete experimental design matrix is shown in Table 3. The pretreatment experiments were performed in a random order.

(1) $$X_{i}=X_{\text{min}}+\left(\left(x_{i}+1\right)/2\right).\left(X_{\text{max}}-X_{\text{min}}\right)\;i=1,\;2,\;....,\;n$$

Where *X*_*i*_ is the uncoded value of the independent variable i, *X*_*min*_ and *X*_*max*_ are the uncoded minimum and maximum values (corresponding to -1 and +1 coded values), and *x*_*i*_ is the code value to be translated.

The second order polynomial model described by Equation 2 was used for predicting the optimal pretreatment conditions.

(2) $$Y=\beta_{0}+\sum_{i-1}^{n}\beta_{i}X_{i}+n\sum_{i-1}^{n}\beta_{\mathrm{ii}}X_{\mathrm{ii}}^{2}+\sum_{i-1}^{n-1}\sum_{j-1}^{n}\beta_{\mathrm{ij}}X_{i}X_{j}$$

Where *Y* is estimated value of the response; *n* is the number of independent variables; β_0_ is an intercept, β_i_, β_ii_ and β_ij_ stand for regression coefficients for linear, quadratic and interaction of two independent variables; *X*_*i*_, $X_{i}^{2}$ and *X*_*i*_*X*_*j*_ refers to as linear, quadratic and two way interaction effects, respectively. Regression analysis was performed by Design Expert, version 8.0.2 (State Ease Inc., Minneapolis, United States). The software also determines which independent variables have significant effects on the process responses by ANOVA.

The equation obtained for the combined sugar yield was used in Matlab 8.1 (R2013a) to construct contour plots representing the pretreatment conditions (temperature and residence time) that provide 95% of the maximum combined sugar yield for each of the bagasses.

### Enzymatic hydrolysis

The WIS fraction of pretreated bagasse samples was subjected to enzymatic hydrolysis to evaluate the effect of the pretreatment and the differences between sugarcane varieties on the hydrolysis of bagasse. These experiments were conducted in 250 ml Erlenmeyer flasks. The flasks were loaded with 1 g (dry weight) of WIS and 50 ml of 0.05 M citrate buffer (pH 4.8) with the enzyme solution, to give a solids loading of 2% (w/v). Sodium azide was added at a concentration of 0.02% (w/v) to prevent microbial growth. Two commercial enzymes preparations were used: Spezyme CP (Genencor-Danisco, Brabrand, Denmark) with cellulase activity of 65 FPU/ml and Novozym 188 (Novozymes A/S, Bagsvaerd, Denmark) with β-glucosidase activity of 995 IU/ml. Enzyme activities were determined according to Ghose [34]. Cellulase loading of 0.2308 mL/ g WIS (15 FPU/g WIS) of Spezyme CP supplemented with β-glucosidase of 0.01508 mL/g WIS (15 IU/g WIS) was applied in all the experiments. Flasks loaded with the mixtures were placed in water bath maintained at 50°C with shaking at 90 rpm. Samples were withdrawn after 72 h and prepared for analysis as described below.

### Yeast and culture medium

*S. cerevisiae* MH1000 was used in the SSF experiments [35]. The yeast strain was stored at -80°C in the presence of 30% glycerol in vials and transferred to agar plates prior to use. The pre-inoculum was grown in a 250 ml Erlenmeyer flask containing 50 ml of mineral media (20 g.l^-1^ yeast extract, 7.5 g.l^-1^ (NH4)_2_SO_4_, 3.4 g.l^-1^ KH_2_PO_4_, 0.8 g.l^-1^ MgSO_4_.7H_2_O, 1 ml trace element solution, 0.05 g.l^-1^ CaCl_2_.H_2_O, 0.5 g.l^-1^ and Citric acid, and 20 g.l^-1^ glucose) [36] for 24 hours at a temperature of 30°C with agitation speed of 150 rpm. A sample of this starting culture was transferred to a 1 L Erlenmeyer flask with 300 ml of preconditioning medium (mineral media with 20% (v/v) of pretreatment liquor) with an initial OD of 0.2. The preconditioning media was incubated at 30°C and 150 rpm until it reached an OD of 4.5-5.5 (approximately after 16 to 18 hours). The preculture was harvested by centrifugation at 8000 rpm for 5 minutes (Model Z366, Hermle Labortechnik GmbH, Wehingen, Germany). The supernatant was discarded and the pellet was washed with phosphate buffered saline (PBS) solution (containing 8.01 g.l^-1^, NaCl; 0.2 g.l^-1^, KCl; 1.78 g.l^-1^, Na_2_HPO_4_.2H_2_O; 0.27 g.l^-1^, KH_2_PO_4_; adjust pH to 7.4 with the addition of 3 M KOH) and centrifuged again. The washing with PBS solution was repeated three times. The final pellet was diluted in PBS to obtain the selected inoculum size to start the SSF (5 g/l wet cells equivalent to 1.34 g/l dry cells).

The media were sterilized by autoclaving while the pretreated liquor was sterile filtered (0.22 μm Stericup, Millipore, Billerica, United States).

### Simultaneous saccharification and fermentation (SSF)

The pressed slurry from optimum pretreatment conditions (maximum combined sugar yield) was used as substrate of SSF experiments. The slurry was pressed to a final moisture content of between 59 and 63% using a 50 ton shop press with gauge model TDR NO. 55002 (Northern Tool and Equipment Company, Minnesota, United States) set at 5 MPa. The SSF was conducted in batch and fed-batch regime. Two loadings of enzymes containing the mixture of Cellic Ctec2 (cellulase) and Cellic Htec2 (endoxylanase) kindly provided by Novozymes (A/S, Bagsvaerd, Denmark) were applied under the batch process but only a single dosage was used for the fed-batch process. These loadings were selected based on enzymatic hydrolysis optimization of sugarcane bagasse obtained from previous study [37]. The densities of Cellic Ctec2 and Cellic Htec2 were 1.09 and 1.22 g.ml^-1^, respectively.

Samples were withdrawn periodically and analyzed in high performance liquid chromatograph (HPLC) for sugars, ethanol, glycerol and byproducts as described below.

### Batch SSF with different enzyme dosage

The batch SSF was performed at a solid loading of 10% (w/w) at 35°C with 150 rpm for five days. Batch SSF were conducted in 250 ml Erlenmeyer flask with a final working weight of 200 g. The unsterilized pressed slurry was supplemented with mineral media without glucose and the pH was adjusted by adding 3 M KOH. After adjustment of pH to 5, Cellic Ctec2 and Cellic Htec2 were added at two loadings. The first loading was 0.15 ml of Cellic Ctec2/g pretreated material (dry basis) and 0.0167 ml of Cellic Htec2/g pretreated material. For the second enzymes loading, Cellic Ctec2 and Cellic Htec2 were added at 0.15 ml/g pretreated material and 0.213 ml/g pretreated material, respectively. After the enzymes were added the mixture was left for 1 hour for pre-saccharification at temperature of 35 ˚C. Thereafter the inoculum was added at a concentration of 5 g/l of wet cells (corresponding to approximately 1.34 g/l dry cells).

### Fed-batch SSF

The fed-batch experiments were conducted in 1 L bioreactor (BioFlo110, New Brunswick Scientific Co., Inc., Canada) with a final working weight of 0.6 kg at a set temperature of 35˚C and 150 rpm. The reactor containing the mineral medium without glucose was autoclaved at 121 ˚C for 15 minutes. The experiment was started by adding the pretreated material (unsterilized) in order to give an initial 2% (w/w) of solids loading. Cellic Ctec2 and Cellic Htec2 were added to give the final enzyme dosage of 0.15 ml/g pretreated material and 0.213 ml/g pretreated material, respectively. The yeast cells were added after one hour of pre-saccharification to give a final concentration of 5 g /l of wet cells. The pH was maintained at 5 by the controlled addition of 3 M KOH. The substrate was loaded twice daily (2% w/w each) until the final loading of 16% was reached.

### Chemical analyses

The carbohydrates and lignin contents of the extract-free raw materials and WIS were determined by the laboratory analytical procedures (LAPs) proposed by the National Renewable Energy Laboratory (NREL) [38-40]. The sugar and by-products concentration of pretreatment liquor were analyzed by HPLC. The pretreatment liquor was subjected to a mild acid hydrolysis to convert the sugars in oligomeric form into monomers [41]. The difference in concentration before and after the hydrolysis was assumed to be in oligomeric form.

Monomeric sugars, acetic acid, formic acid, ethanol and glycerol were determined by HPLC system equipped with an Aminex HPX-87H Column and a Cation-H Micro-Guard Cartridge (Bio-Rad, Johannesburg, South Africa). The column was set to a temperature of 65°C with a mobile phase of 5 mM sulfuric acid and a flow rate of 0.6 ml/min. The concentrations were measured with a RI detector (Shodex, RI-101, Munich, Germany) operated at 45°C. Since xylose and galactose, and mannose-arabinose co-eluted in the H-column, additional HPLC analysis were conducted. For these analysis the HPLC system was equipped with an Xbridge^TM^ Amide column (Waters Corporation, Massachusetts, United States) (4.6 × 250 mm, 3.5 μm particle size) and a Xbridge™ Amide precolumn (Waters) set at 30°C using 0.05% ammonium hydroxide in water (A) and 0.05% ammonium hydroxide in 90% acetonitrile (B) as mobile phase with a flow rate of 0.7 ml/min. Sugars were detected by a Varian 380-LC evaporative light-scattering detector (Agilent Technologies, California, United States). Since galactose and mannose contents were minimal, the quantification provided by the Aminex HPX-87H column was considered accurate.

The glucan, xylan, arabinan, o-acetyl group contents in raw material and WIS were determined by applying a conversion factor: as (0.95 × cellobiose + 0.9 × glucose), 0.88 × xylose, 0.88 × arabinose and 0.683 × acetic acid, respectively [39].

The concentration of HMF and furfural in the pretreated liquor were analyzed on a Phenomenex Luna C18(2) reversed phase column equipped with a Phenomenex Luna C18(2) precolumn (Separations, Johannesburg, South Africa) with column temperature set to 25°C and a flow rate of 0.7 ml/min. The mobile phases used for elution were 5 mM trifluoroacetic acid in water (A) and 5 mM trifluoroacetic acid in acetonitrile (B). Separation was carried out by gradient elution from 5% mobile phase B, increasing to 11% B over 14 minutes and then increasing to 40% B over 3 minutes. The mobile phase composition was then kept constant at 40% for 2 minutes, followed by a decrease to 5% B over 5 minutes and ending with a final step of constant composition at 5% B for 4 minutes in order to equilibrate. HMF and furfural concentrations were measured with a Dionex Ultimate 3000 diode array detector (Thermo Fisher Scientific, Calfornia, United States) at 215 nm and 285 nm.

### Statistical analysis, severity and ethanol calculation

The chemical compositions of untreated materials were calculated as the average values and standard deviations (average ± SD). One-Way-ANOVA was employed to evaluate the statistical significance of yield differences between various bagasse samples. The hypothesis was accepted or rejected at 95% confidence interval. The combined severity factor (CSF = log*Ro’*) was calculated based on Equation 3 [42] while ethanol yield per hectare was estimated according to Equation 4.

(3) logR'0=logt.expTH-10014.75-pHout

Where, ‘*t*’ is reaction time in minutes, ‘*T*_*H*_’ is the reaction temperature in°C, 100 is the reference temperature, ‘pH_out_’ is the pH of the pretreated liquor.

(4) $$E_{Y}=X\times C_{Y}\times T_{C}\times \eta_{S}\times \left(1000/\rho_{E}\right)$$

Where *E*_*Y*_, ethanol yield (L.ha^-1^); *X,* is sugars content in both juice and bagasse (%cane); *C*_*Y*_ is the cane yield (ton.ha^-1^); *T*_*C*_, stoichiometric conversion factor (0. 538 for sucrose and 0.5111 for other sugars); *σ*_*s*_, conversion efficiency of substrate to ethanol when the fermentation employs MH1000 yeast strain; and 1000/0.789 (density of ethanol at 20°C, g.ml^-1^).

## Abbreviations

AIS: acid insoluble lignin; CCD: central composite design; CSY: combined sugar yield; FPU: filter paper unit; HMF: hydroxymethylfurfural; HPLC: high-performance liquid chromatography; LAPs: laboratory analytical procedures; SASRI: South Africa sugarcane research institute; SSF: simultaneous saccharification and fermentation; WIS: water insoluble solid; XOSs: xylo-oligomers, RM, raw material, ha, hectare; ANOVA: analysis of variance.

## Competing interests

The authors declare that they have no competing interests.

## Authors’ contributions

YB participated in designing of the study, performed out all experiments, data interpretation and drafted the manuscript. MPG participated in designing of the study and reviewed the manuscript. JFG conceived the study, participated in its design coordination and reviewed the manuscript. All authors read and approved the final manuscript.

## References

[B1] Waclawovsky AJ Sato PM Lembke CG Moore PH Souza GM Sugarcane for bioenergy production: an assessment of yield and regulation of sucrose content Plant Biotechnol J 2010 8 263 276 20388126 10.1111/j.1467-7652.2009.00491.x

[B2] Somerville C Youngs H Taylor C Davis SC Long SP Feedstocks for lignocellulosic biofuels Science 2010 329 790 792 20705851 10.1126/science.1189268

[B3] Dias MO Junqueira TL Cavalett O Cunha MP Jesus CD Rossell CE Filhoa RM Bonomia A Integrated versus stand-alone second generation ethanol production from sugarcane bagasse and trash Bioresour Technol 2012 103 152 161 22019267 10.1016/j.biortech.2011.09.120

[B4] Sun Y Cheng J Hydrolysis of lignocellulosic materials for ethanol production: a review Bioresour Technol 2002 83 1 11 12058826 10.1016/s0960-8524(01)00212-7

[B5] Wyman CE What is (and is not) vital to advancing cellulosic ethanol TRENDS Biotechnol 2007 25 153 157 17320227 10.1016/j.tibtech.2007.02.009

[B6] Benjamin Y Cheng H Görgens JF Evaluation of bagasse from different varieties of sugarcane by dilute acid pretreatment and enzymatic hydrolysis Ind Crops Prod 2013 51 7 18

[B7] Sassner P Galbe M Zacchi G Techno-economic evaluation of bioethanol production from three different lignocellulosic materials Biomass Bioenergy 2008 32 422 430

[B8] Zhang J Chu D Huang J Yu Z Dai G Bao J Simultaneous saccharification and ethanol fermentation at high corn stover solids loading in a helical stirring bioreactor Biotechnol Bioeng 2010 105 718 728 19882718 10.1002/bit.22593

[B9] Kim Y Mosier NS Ladisch MR Ramesh Pallapolu V Lee YY Garlock R Balan V Dale BE Donohoe BS Vinzant TB Elander RT Falls M Sierra R Holtzapple MT Shi J Ebrik MA Redmond T Yang B Wyman CE Warner RE Comparative study on enzymatic digestibility of switchgrass varieties and harvests processed by leading pretreatment technologies Bioresour Technol 2011 102 11089 11096 21741233 10.1016/j.biortech.2011.06.054

[B10] Kučerová J The effect of year, site and variety on the quality characteristics and bioethanol yield of winter triticale J Inst Brew 2007 113 142 146

[B11] Jakob K Zhou F Paterson A Genetic improvement of C4 grasses as cellulosic biofuel feedstocks Vitro Cell Dev Biol-Plant 2009 45 291 305

[B12] Dien B Sarath G Pedersen J Sattler S Chen H Funnell-Harris D Nichols NN Cotta MA Improved sugar conversion and ethanol yield for forage sorghum (Sorghum bicolor L. Moench) lines with reduced lignin contents BioEnergy Res 2009 2 153 164

[B13] Masarin F Gurpilhares DB Baffa DCF Barbosa MHP Carvalho W Ferraz A Milagres AMF Chemical composition and enzymatic digestibility of sugarcane clones selected for varied lignin contents Biotechnol Biofuel 2011 4 55 64 10.1186/1754-6834-4-55 PMC3267660 22145819

[B14] Lindedam J Andersen SB DeMartini J Bruun S Jørgensen H Felby C Magid J Yang B Wyman CE Cultivar variation and selection potential relevant to the production of cellulosic ethanol from wheat straw Biomass Bioenergy 2012 37 221 228

[B15] Isci A Murphy PT Anex RP Moore KJ A rapid simultaneous saccharification and fermentation (SSF) technique to determine ethanol yields BioEnergy Res 2008 1 163 169

[B16] Torres AF van der Weijde T Dolstra O Visser RGF Trindade LM Effect of maize biomass composition on the optimization of dilute-acid pretreatments and enzymatic saccharification BioEnergy Res 2013 6 1038 1051

[B17] Jung JH Fouad WM Vermerris W Gallo M Altpeter F RNAi suppression of lignin biosynthesis in sugarcane reduces recalcitrance for biofuel production from lignocellulosic biomass Plant Biotechnol J 2012 10 1067 1076 22924974 10.1111/j.1467-7652.2012.00734.x

[B18] Watt DA Sweby DL Potier BAM Snyman SJ Sugarcane genetic engineering research in South Africa: from gene discovery to transgene expression Sugar Tech 2010 12 85 90

[B19] Kabel MA Bos G Zeevalking J Voragen AG Schols HA Effect of pretreatment severity on xylan solubility and enzymatic breakdown of the remaining cellulose from wheat straw Bioresour Technol 2007 98 2034 2042 17029957 10.1016/j.biortech.2006.08.006

[B20] Ramos LP The chemistry involved in the steam treatment of lignocellulosic materials Quim Nova 2003 26 863 871

[B21] Canilha L Santos VTO Rocha GJM de Silva JBA Giulietti M Silva SS Felipe MGA Ferraz A Milagres AMF Carvalho W A study on the pretreatment of a sugarcane bagasse sample with dilute sulfuric acid J Ind Microbiol Biotechnol 2011 38 1467 1475 21210180 10.1007/s10295-010-0931-2

[B22] Olofsson K Bertilsson M Lidén G A short review on SSF-an interesting process option for ethanol production from lignocellulosic feedstocks Biotechnol Biofuel 2008 1 1 14 10.1186/1754-6834-1-7 PMC2397418 18471273

[B23] Pienkos PT Zhang M Role of pretreatment and conditioning processes on toxicity of lignocellulosic biomass hydrolysates Cellulose 2009 16 743 762

[B24] Kasemets K Kahru A Laht T-M Paalme T Study of the toxic effect of short-and medium-chain monocarboxylic acids on the growth of saccharomyces cerevisiae using the CO_2_-auxo-accelerostat fermentation system Int J Food Microbiol 2006 111 206 215 16945441 10.1016/j.ijfoodmicro.2006.06.002

[B25] Galbe M Zacchi G A review of the production of ethanol from softwood Appl Microbiol Biotechnol 2002 59 618 628 12226717 10.1007/s00253-002-1058-9

[B26] Bezuidenhout CN Singels A Operational forecasting of South African sugarcane production: part 2 – system evaluation Agric Syst 2007 92 39 51

[B27] Liang L Zhang Y Zhang L Zhu M Liang S Huang Y Study of sugarcane pieces as yeast supports for ethanol production from sugarcane juice and molasses J Ind Microbiol Biotechnol 2008 35 1605 1613 18685877 10.1007/s10295-008-0404-z

[B28] Wahlbom CF van Zyl WH Jönsson LJ Hahn-Hägerdal B Otero RRC Generation of the improved recombinant xylose-utilizing saccharomyces cerevisiae TMB 3400 by random mutagenesis and physiological comparison with Pichia stipitis CBS 6054 FEMS Yeast Res 2003 3 319 326 12689639 10.1016/S1567-1356(02)00206-4

[B29] Benjamin Y Cheng H Görgens JF Optimization of dilute sulfuric acid pretreatment to maximize combined sugar yield from sugarcane bagasse for ethanol production Appl Biochem Biotechnol 2014 172 610 630 24104688 10.1007/s12010-013-0545-z

[B30] Dien BS Jung H-JG Vogel KP Casler MD Lamb JFS Iten L Mitchell RB Sarath G Chemical composition and response to dilute-acid pretreatment and enzymatic saccharification of alfalfa, reed canarygrass, and switchgrass Biomass Bioenergy 2006 30 880 891

[B31] Guo G-L Chen W-H Chen W-H Men L-C Hwang W-S Characterization of dilute acid pretreatment of silvergrass for ethanol production Bioresour Technol 2008 99 6046 6053 18262784 10.1016/j.biortech.2007.12.047

[B32] Chang VS Holtzapple MT Fundamental factors affecting biomass enzymatic reactivity Appl Biochem Biotechnol 2000 84–86 5 37 10.1385/abab:84-86:1-9:5 10849776

[B33] Sluiter A Hames B Hyman D Payne C Ruiz R Scarlata C Sluiter J Templeton D Wolfe J Determination of total solids in biomass and total dissolved solids in liquid process samples Laboratory analytical procedure (LAP): NREL/TP-510-42621 2008 Golden, Colorado: National Renewable Energy Laboratory

[B34] Ghose TK Measurement of cellulase activities Pure Appl Chem 1987 59 257 268

[B35] van Zyl JM van Rensburg E van Zyl WH Harms TM Lynd LR A kinetic model for simultaneous saccharification and fermentation of avicel with saccharomyces cerevisiae Biotechnol Bioeng 2011 108 924 933 21404265 10.1002/bit.23000

[B36] Verduyn C Postma E Scheffers WA Van Dijken JP Effect of benzoic acid on metabolic fluxes in yeasts: a continuous-culture study on the regulation of respiration and alcoholic fermentation Yeast 1992 8 501 517 1523884 10.1002/yea.320080703

[B37] Wallace J Enzymatic hydrolysis of steam pretreated bagasse: enzyme preparations for efficient cellulose conversion and evaluation of physiochemical changes during hydrolysis 2013 Stellenbosch University, Process Engineering Department: MSc Thesis

[B38] Sluiter A Hames B Ruiz R Scarlata C Sluiter J Templeton D Determination of ash in biomass Laboratory analytical procedure (LAP): NREL/TP-510-42622 2008a Golden, Colorado: National Renewable Energy Laboratory

[B39] Sluiter A Hames B Ruiz R Scarlata C Sluiter J Templeton D Crocker D Determination of structural carbohydrates and lignin in biomass Laboratory analytical procedure (LAP): NREL/TP-510-42618 2008b Golden, Colorado: National Renewable Energy Laboratory

[B40] Sluiter A Ruiz R Scarlata C Sluiter J Templeton D Determination of extractives in biomass Laboratory analytical procedure (LAP): NREL/TP-510-42619 2005 Golden, Colorado: National Renewable Energy Laboratory

[B41] Sluiter A Hames B Ruiz R Scarlata C Sluiter J Templeton D Determination of sugars, byproducts, and degradation products in liquid fraction process samples Laboratory analytical procedure (LAP): NREL/TP-510-42623 2008c Golden, Colorado: National Renewable Energy Laboratory

[B42] Chum HL Johnson DK Black SK Overend RP Pretreatment-catalyst effects and the combined severity parameter Appl Biochem Biotechnol 1990 24–25 1 14

